# Case report of Pierre Robin sequence with severe upper airway obstruction who was rescued by fiberoptic nasotracheal intubation

**DOI:** 10.1186/s12871-017-0336-0

**Published:** 2017-03-14

**Authors:** Satoru Takeshita, Hiroko Ueda, Tatenobu Goto, Daisuke Muto, Hiroki Kakita, Kazuo Oshima, Takahisa Tainaka, Takayuki Ono, Yoshiaki Kazaoka, Yasumasa Yamada

**Affiliations:** 1 Perinatal and Neonatal Center, Aichi Medical University, 1-1 Yazakokarimata, Nagakute, 480-1195 Aichi Japan; 2 Department of Pediatric Surgery, Nagoya University Graduate School of Medicine, 65 Tsurumai-cho, Showa-ku, Nagoya, 466-8560 Aichi Japan; 3 Oral Surgery Department, Aichi Medical University, 1-1 Yazakokarimata, Nagakute, 480-1195 Aichi Japan

**Keywords:** Pierre Robin sequence, Micrognathia, Glossoptosis, Airway obstruction, Fiberoptic nasotracheal intubation, Case report

## Abstract

**Background:**

Pierre Robin sequence (PRS) refers to the association of micrognathia, glossoptosis, and airway obstruction. Cases with severe dyspnea due to upper airway obstruction immediately after birth are very rare. We here report two cases with PRS who developed severe dyspnea due to morphological abnormality immediately after birth and were rescued by fiberoptic nasotracheal intubation.

**Case presentation:**

The patient in case 1 had micrognathia and cleft palate, and his tongue protruded into the nasal cavity via a cleft palate. His invaginated tongue was considered an extreme type of glossoptosis and he was diagnosed as Pierre Robin sequence. The patient in case 2 also had micrognathia and cleft palate same as case 1 and accompanied some anomalad. Her chromosome analysis confirmed a diagnosis of 1p36 deletion syndrome and she diagnosed as 1p36 deletion syndrome complicated with Pierre Robin sequence. In both cases, tongue protruded into the nasal cavity via a cleft palate occupied pharynx and nasal cavity, resulting in severe dyspnea. Only the backside of the tongue was visible by laryngoscopy and oropharyngeal intubation was impossible. Therefore, fiberoptic nasotracheal intubation was done to secure the airway for resuscitation.

**Conclusion:**

We conclude that extreme type of glossoptosis in PRS concludes tongue invaginated into nasal cavity which have not reported before and that such cases require resuscitation by fiberoptic intubation immediately after birth. As such, neonatologists should obtain the skill of fiberoptic intubation.

## Background

Pierre Robin sequence (PRS) has been described as a triad of micrognathia, glossoptosis, and upper airway obstruction, which occurs in 1/8500 to 1/14,000 live births and is often accompanied by cleft palate [1]. PRS often develops upper airway obstruction or feeding difficulty secondary to micrognathia, glossoptosis, or a shifted tongue that comes in contact with the pharyngeal wall [2]. Usually, progressive airway obstruction might become more noticeable in the second month of life. Cases with severe dyspnea due to upper airway obstruction immediately after birth are very rare. Here, we report two PRS cases with severe airway obstruction that was rescued by fiberoptic nasotracheal intubation. In this report, CARE guidelines/methodology was adhered to.

## Case presentation

### Case 1

**Fig. 1:**
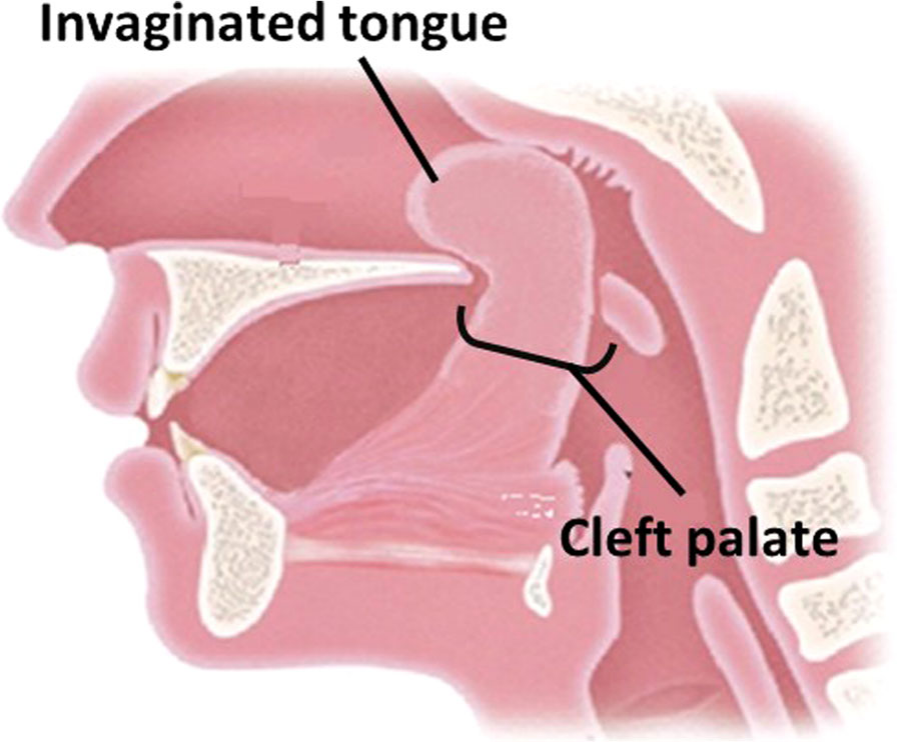
Schema of larynx in case 1 is shown. The tongue of patient protrudes to nasal cavity via cleft palate, resulting in tongue occupying not only pharynx but also nasal airway, which causes critical dyspnea immediately at birth

**Fig. 2:**
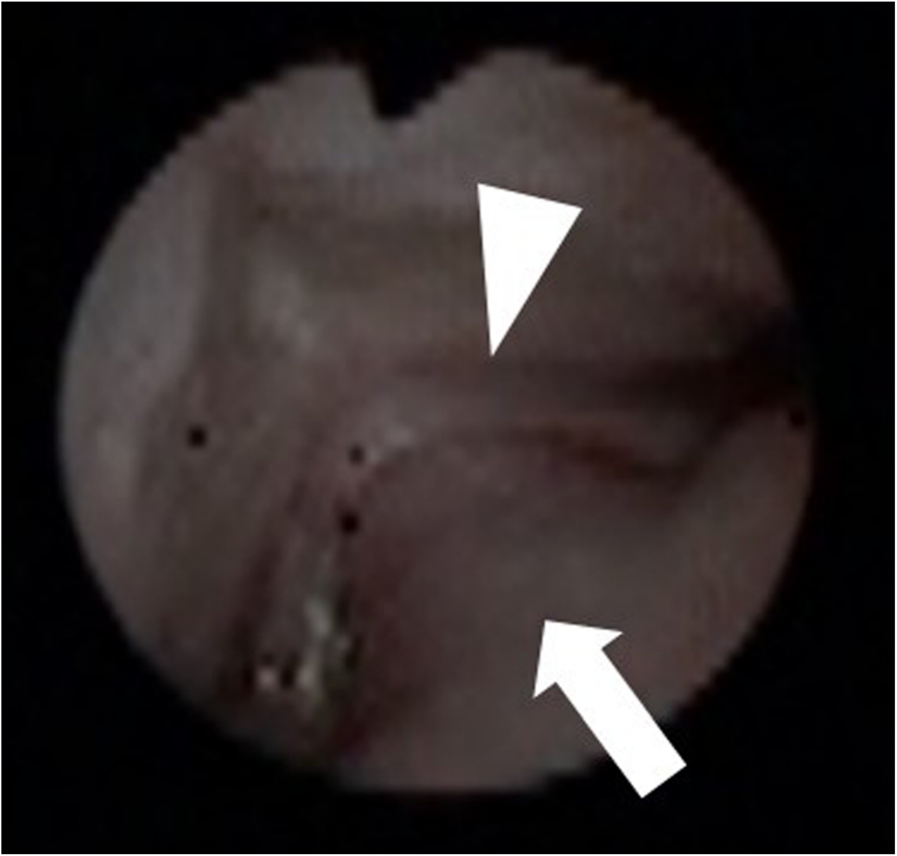
Imaging finding of the oral cavity during fiberoptic intubation in Case 1. Cleft palate (*triangle*): root of the tongue (*arrowhead*). The tongue protruding into the nasal cavity via a cleft palate results in larynx not visible

A 2574 g boy was born at 36 weeks gestational age by normal vaginal delivery after an uncomplicated pregnancy. No family history of congenital disorders was present. He developed severe dyspnea immediately after birth. He had micrognathia and cleft palate and his tongue protruded into the nasal cavity via a cleft palate; as a result, only the backside of the tongue was visible by laryngoscopy (Figs. 1 and 2). Other physical examination findings were normal. Airway was completely obstructed, and the obstruction was not relieved by any change of position. Manual ventilation with high concentration oxygen or the use of nasopharyngeal airway was ineffective, and oropharyngeal intubation was impossible. Therefore, fiberoptic nasotracheal intubation was finally performed to secure the airway for resuscitation. The endoscope fiber was 2.8 mm in outer diameter, and the intubation tube was 3.0 mm in inner diameter. Intubation was performed in supine position, and we carefully inserted a videoscope via a small interstice between the invaginated tongue and the pharyngeal wall. Before and during intubation, severe hypoxia (SpO2 < 80%) lasted, and insufficient spontaneous breathing with high concentration oxygen supply was only available treatment. Because the urgent situation allowed us no time to prepare drugs, no sedatives were given. During the procedure of nasotracheal intubation, no complications or problems other than severe hypoxia were observed. This clinical condition was considered an extreme type of glossoptosis and he was diagnosed as PRS. Subsequently, he underwent tracheostomy and is now under medical treatment at home.

### Case 2

A 2282 g girl was born at 37 weeks gestational age by normal vaginal delivery. No family history of congenital disorders was present. Pre-delivery fetal ultrasound showed polyhydramnios. She developed severe dyspnea immediately after birth. She had micrognathia and cleft palate; her tongue protruded to the nasal cavity via the cleft palate, which resulted in only the backside of the tongue appearing on laryngoscopy. Her laryngoscopic appearance was similar to that of Case 1. Manual ventilation or oral intubation was impossible and fiberoptic nasotracheal intubation was done for resuscitation as same as case 1. Her invaginated tongue was also considered an extreme type of glossoptosis and she was diagnosed as PRS. Additionally, she had low anorectal anomaly, severe hearing loss, and hypoplastic auricle. Chromosome analysis confirmed a diagnosis of 1p36 deletion syndrome. Subsequently, she underwent tracheostomy and is now under medical treatment at home.

## Discussion

We reported two PRS cases that developed severe upper airway obstruction immediately after birth and were rescued by fiberoptic nasotracheal intubation. PRS refers to the association of micrognathia and glossoptosis and is characterized by varying degrees of upper airway obstruction, due mainly to glossoptosis. Usually, progressive airway obstruction might become more noticeable in the second month of life [2]. In our cases, micrognathia and an extreme type of glossoptosis resulted in severe airway obstruction and difficult oropharyngeal intubation at birth. Based on fiberoptic endoscopy, mechanism of upper airway obstruction in PRS is classified into four types according to position of tongue or palate attaching to pharyngeal wall [3]. None of the four types in this classification was applicable to a novel type of our cases. Extreme posterior shifting of the tongue is considered an obstacle in fusion of the bilateral palate in the early fetal stage, resulting in a cleft palate. In these cases, the tongue protruding into the nasal cavity occupied not only the pharynx, but also the nasal airway, resulting in severe dyspnea. To our knowledge, there have been no reports on PRS patients experiencing severe dyspnea immediately after birth due to critical airway obstruction and who were rescued by fiberoptic nasotracheal intubation.

Case 2 had a diagnosis of 1p36 deletion syndrome with low anorectal anomaly, severe hearing loss, and hypoplastic auricle, in addition to the airway anomalies. 1p36 deletion syndrome affects approximately 1 in 5000 newborns and is the most common terminal chromosomal deletion in humans [4]. Medical problems commonly caused by this anomaly include developmental delay, intellectual disability, seizures, vision loss, short stature, distinctive facial features, brain anomalies, orofacial clefting, congenital heart defects, and renal anomalies. Case 2 had 1p36 deletion syndrome complicated with PRS. Guidelines for difficult airway management in neonatal cardiopulmonary resuscitation have not been established. Fiberoptic intubation is the gold standard for pediatric difficult airways. However, the American Society of Anesthesiologists suggested that during anesthetic induction for emergent tracheotomy, fiberoptic intubation is the second choice for patients in which oropharyngeal intubation is difficult [5]. The anatomical features of infants include a cephalad larynx, fragile epiglottis, and a large tongue relative to the oropharyngeal space [6]. Fiberoptic intubation allows a more straightforward pathway to the larynx and can compensate for these features. In our cases, it was difficult to predict the occurrence of severe upper airway obstruction before birth because fetal ultrasound findings were normal, except for polyhydramnios in Case 2. For cases in which airway compromise at birth cannot be predicted, preparation and acquisition of fiberoptic intubation equipment may be necessary for neonatologists.

## Conclusion

We conclude that extreme type of glossoptosis in PRS concludes tongue invaginated into nasal cavity which have not reported before and that such cases require resuscitation by fiberoptic intubation immediately after birth. As such, neonatologists should obtain the skill of fiberoptic intubation.

## Abbreviation

PRS: Pierre Robin sequence
